# Long-Lasting Insecticidal Nets for Malaria Control in Myanmar and Nigeria: Lessons From the Past, Tools for the Future

**DOI:** 10.9745/GHSP-D-18-00158

**Published:** 2018-06-27

**Authors:** Michael B. Macdonald

**Affiliations:** a Independent consultant, Catonsville, MD, USA.

## Abstract

While having saved many lives over the past decade, continued dependence on mass distribution of free long-lasting insecticidal nets (LLINs) is not sufficient and may not be sustainable. Programs must be enabled with flexible policy and technical options to place LLINs within a larger context of multisectoral partnerships and integrated vector management, avoiding what happened in the DDT era, where there was overreliance implementing a uniform solution to a complex problem.

See also related articles by Acosta and by Kheang.

Two articles appearing in this issue of GHSP illustrate the diverse challenges—and limitations—of malaria vector control that has become heavily reliant on mass distributions of free insecticide-treated nets (ITNs). On the surface, the 2 contexts could not seem more different. Acosta et al. describe a school-based distribution scheme in Cross River State, Nigeria,^1^ and Kheang et al.^2^ service delivery strategies for mobile and migrant populations in Myanmar: two vastly different scenarios with unique *tactical* problems, but with options limited by the common *strategic* solution being implemented.

Mass distribution of free ITNs has shown proven success across many contexts. Between 2000 and 2015, reported malaria cases dropped from 271 million to 212 million, and deaths from 864,000 to 429,000^3^; 68% of the decline was attributed to ITNs, 19% to availability of artemisinin-based combination therapy, and 13% to indoor residual spraying (IRS).^4^ However, according to the latest data from the World Health Organization (WHO), there were an estimated 216 million cases of malaria in 2016, marking a return to 2012 levels, and deaths stood at about 445,000, similar to the previous year.^5^ Speaking at a Malaria Summit during the recent 2018 Commonwealth Heads of Government Meeting in London, Dr. Tedros Ghebreyesus, WHO Director-General, said^6^:

*The latest data show that we are now at crossroads. If we relax our efforts, we know that malaria will come roaring back—and with a vengeance.*

Dr. Pedro Alonzo, Director of the WHO Global Malaria Programme, struck a similar note in a World Malaria Day interview^7^:

*We are at a real crossroads. We've seen great progress, we're now stalling. Why are we stalling? Possibly because funding has plateaued for the last five or six years. With population growth, that means that per capita investment in the fight against malaria is decreasing in a great number of countries, and we haven't had any new transformative tools come onto the market so it doesn't seem hard to imagine that with the same level of funding, with the same tools we are seeing the limit to what we can do.*

If we are to succeed in our malaria elimination efforts, we need to remember the words of Dr. José Nájera, former director of the WHO malaria program^8^:

*Before DDT, malariologists were trained as problems solvers, after DDT, malariologists were trained as solution implementors.*

DDT (dichlorodiphenyltrichloroethane) was the first modern synthetic insecticide that initially had great success and broad use but was later greatly reduced after evidence emerged that its benefits were declining due to development of resistance by many insect species and because of its harmful environmental effects. We need to move beyond the current “solution” of exclusive reliance on public-sector free mass LLIN distributions. Instead, under the context of integrated vector management that recognizes that effective vector control is not the sole preserve of the health sector but requires the collaboration of various public and private agencies and community participation,^9^ we should enable program managers to engage all available resources to “solve the problem” of malaria vector control in their unique circumstances.

We need to enable program managers to engage all available resources to “solve the problem” of malaria vector control in their unique circumstances.

## A SIMPLE SOLUTION TO A COMPLEX PROBLEM

ITN distribution strategies narrowed for several years and are just now modestly expanding. Prior to support from the Global Fund to Fight AIDS, Tuberculosis and Malaria, the WHO/Roll Back Malaria ITN distribution strategy focused on building partnerships among the public sector, the commercial sector, and NGOs, as illustrated in the Figure.^10^ Considerable efforts were made through the United States Agency for International Development-supported NetMark project, the UK Department for International Development (DfID), the Canadian International Development Research Centre (IDRC), the Swiss Agency for Development and Cooperation, and others to develop these partnerships and strategies, with one of the larger examples being the Tanzania National Voucher Scheme.^11^

**FIGURE:**
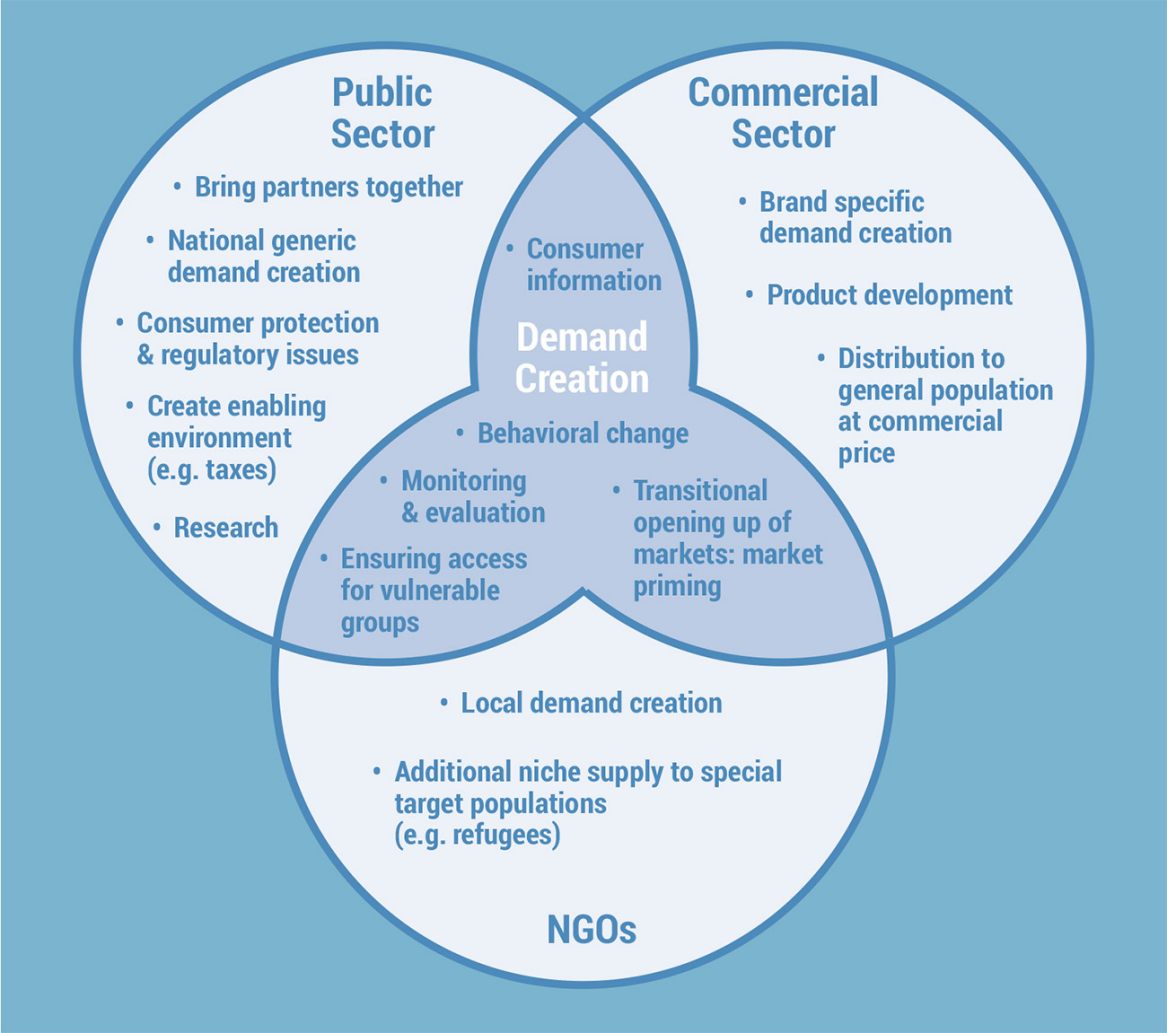
Roles of the Public Sector, NGOs, and the Commercial Sector in Creating an Enabling Environment for ITN Distribution Abbreviation: ITN, insecticide-treated net. Source: WHO (2003).^10^

In the mid-2000s, two things happened. First, there was recognition that just protecting “vulnerable groups” (i.e., pregnant women and children and people living with HIV/AIDS) had less impact than “universal coverage” for all populations at risk and that in addition to personal protection, ITNs would have a “mass effect” and reduce vectoral capacity of the local mosquito vector population.^12^

Second, the expansion of resources through the Global Fund and the President's Malaria Initiative (PMI), and examples from Cambodia and Ghana of free mass distribution campaigns linked with immunization campaigns, shifted strategies toward exclusive reliance on public-sector distribution.^13^ Now with the commercial sector as vendor rather than partner, the focus became producing uniform ITNs, and later long-lasting insecticidal nets (LLINs), that were able to meet minimum specifications at the lowest possible price in order to win the large tenders for mass distribution. This limited opportunities for product innovation that could add even the most modest price per unit to the LLIN. Moreover, this created a profound division between local commercial net makers and retailers and large producers for the international procurement market.

After the initial waves of episodic free mass LLIN distributions, the need for “between-campaign” continuous distributions became apparent. The 2017 WHO guidance considers antenatal care (ANC), Expanded Programme on Immunization (EPI), and other child health clinics as high-priority continuous LLIN distribution channels where these services are used by a large proportion of the population at risk of malaria. The guidance makes brief mention of private- and commercial-sector channels “as long as these are well-regulated to ensure product quality in line with WHO recommendations.”^14^

After the initial waves of episodic free mass LLIN distributions, the need for “between campaign” continuous distributions became apparent.

## EXPANDING PUBLIC-SECTOR CHANNELS FOR CONTINUOUS DISTRIBUTION

Working within these constraints of centrally procured LLINs distributed through the public sector, Acosta and her team in Nigeria lay out a school-based distribution scheme to 4 grade levels, in addition to the standard ANC distribution, to maintain equitable coverage of nearly 80% of households with at least ITN for the 3 years post-campaign in 3 local government areas (LGAs) of Cross River State, Nigeria. A comparison LGA with only ANC distribution showed a steady decline from 64% to 43% despite availability of ITNs thorough ANC services. The school-based distribution in addition to the standard ANC distribution provided a potential replacement for subsequent mass campaigns in this setting with high rates of school enrollment. Expanding to additional grades may increase the proportion of households with access to a net.

## LLINS, NECESSARY BUT NOT SUFFICIENT

Myanmar presents a different problem. Here, the at-risk population described by Kheang et al. are mobile and migrant workers entering forested areas where they are exposed to the highly efficient malaria vectors *Anopheles dirus* and *Anopheles minimus.* There are several challenges. Many of the mobile population are new to the area with limited knowledge of malaria prevention as well as of public health services in the area. Moreover, some may be engaged in illegal forest activities and reluctant to approach government services. Some work at night or sleep in small temporary shelters where ITNs and IRS are not practical. Kheang found that village malaria workers, mobile teams, and screening points each have strengths and weaknesses in access, cost, and efficiency, and there needs to be a combination of approaches to engage these hard-to-reach populations.

While in general ITNs are effective for malaria prevention in the Greater Mekong Subregion^15^ and free mass ITN distributions in settled villages continue,^16^ there is a long tradition of net use, albeit with untreated nets from the market. The 2016 Myanmar Demographic and Health Survey found that although 97% of households possessed a mosquito net, the vast majority were untreated nets from the market; only 19% of children under 5 and 18% of pregnant women slept under an ITN the previous night.^17^ Cambodia piloted a project for “bundling” untreated nets with insecticide treatments at the wholesale level,^18^ but the IconMaxx and K-O Tab 1-2-3 formulations are no longer being produced. Vietnam continues insecticide retreatment campaigns for the untreated conventional nets, but this is not currently practiced by other programs in the region that continue to rely strictly on standard free mass distribution.^19^

“Outdoor transmission” among mobile and migrant populations present additional challenges. There is nascent work on supplementary vector control tools, including treated shelters, treated clothing and blankets, and topical and spatial repellents.^20^ Recognizing that the market for these new tools may be too small and unstable for serious industry investment, there is an initiative through Roll Back Malaria to link these needs to the much larger needs for vector control in humanitarian emergencies where ITNs and IRS may not be practical.^21^

Taken together, the 2 examples from Myanmar and Nigeria illustrate the risk and limitations of reliance on mass distribution of free LLINs. Cross River State in Nigeria has the advantage of high school attendance enabling an additional channel for continuous distribution. Still, the “solution” remains solely within the public sector and entirely dependent on continued donor support. Risk of supply chain failure can be mitigated through a reconsideration of the partnerships developed in the early days of ITNs, before the Global Fund and PMI, and as recommended in the Multisectoral Action Framework for Malaria.^22^ The LLIN distribution should also be seen in the context of integrated vector management, where larval source management, housing improvements, IRS where appropriate, and new tools such as attractive targeted sugar baits may play a role, including for insecticide resistance management.^23^ Myanmar has these challenges, and more. Insecticide treatment strategies for the commonly used untreated market nets and new tools for “outdoor transmission” beyond the reach of traditional ITNs and IRS are required. Linked to the needs for vector control in humanitarian emergencies, there is growing interest and investment that we hope will enable some of these new tools to come to the market.^24^

LLIN distribution should be seen in the context of integrated vector management.

## LESSONS FROM THE PAST, TOOLS FOR THE FUTURE

As Dr. Tedros and Dr. Alonzo said, we are now at a “crossroads” in malaria vector control. Implementing the current “solution” of mass distribution of free LLINs is not sufficient and may not be sustainable. To reduce risk and optimize use of available resources, we need to learn from the past and revitalize the partnerships and the strategies for multisectoral actions and integrated vector management. We also need to look to the future, beyond LLINs, for tools and processes to prevent outdoor transmission, for the mobile forest worker in Myanmar as well as for displaced families throughout much of the malaria-endemic world. As Dr. Nájera suggests, we need to enable programs with the flexible policy and technical options to move from being “solution implementors” to “problem solvers.”^8^

We are now at a crossroads in malaria vector control whereby the current “solution” of mass distribution of free LLINs is not sufficient and may not be sustainable.

## Notes

**First Published Online:** June 6, 2018
